# Cognitive Behavioural and Art-based program (CB-ART): a pilot study in an early parenting centre

**DOI:** 10.1186/s40814-023-01297-z

**Published:** 2023-08-09

**Authors:** Hilary Brown, Jane Fisher, Julie Cwikel, Orly Sarid, Heather Rowe

**Affiliations:** 1 School of Public Health & Preventive Medicine, Monash University, Melbourne, Australia; 2 Mental Health, Drug and Alcohol Service, Barwon Health, Geelong, Australia; 3 Centre for Women’s Health Studies, Department of Social Work, Ben Gurion University of the Negev, Beer Sheva, Israel

**Keywords:** Art therapy, Cognitive-behavioural intervention, Visual art, Drawing, Postnatal, Pregnancy

## Abstract

**Background:**

The period of pregnancy and early motherhood is a substantial life change associated with psychological turbulence. During this period, some women experience symptoms of anxiety and depression of sufficient severity to warrant professional psychological assistance. Psychosocial and psychological interventions are key therapeutic approaches for women at this life stage. There is growing evidence of the value of the arts in the prevention and treatment of mental health problems. Evidence suggests that women prefer psychological interventions that provide social support and shared space for reflection.

Cognitive Behavioural and Art-based intervention (CB-ART) is a novel therapy for prevention and treatment of perinatal mental health problems. The aim of this study was to implement and evaluate CB-ART for acceptability, feasibility, safety and preliminary efficacy among women admitted to a residential early parenting unit.

**Methods:**

The pilot study used a single-centre, mixed-methods pre- and post-test design to evaluate CB-ART among women admitted to a 5-day residential early parenting service in Melbourne, Australia. Participants completed questionnaires before and after attendance at two 1-h CB-ART group sessions on day 2 and day 5 of admission during which field notes were taken. Evaluation interviews were conducted by telephone 1 week after discharge. The Short Profile of Emotional Competence and the Edinburgh Postnatal Depression Scale were used to assess emotional insight and symptoms of depression, respectively. Feasibility, acceptability and safety were assessed using an analysis of field notes, with quantitative data collected by telephone questionnaire and qualitative data by telephone interviews.

**Results:**

Nine participants enrolled in the program; eight provided complete data. Two CB-ART groups were conducted. Before and after comparisons showed that there was an improvement in symptoms of postnatal depression and a marginal improvement in emotional insight. Thematic analysis of qualitative data indicated CB-ART was a feasible and acceptable means of assisting reflection.

**Conclusion:**

The preliminary data indicate that the CB-ART program is a feasible, acceptable and safe addition to the 5-day residential program, with potentially therapeutic benefits. A larger randomised study is required to assess the effects of the CB-ART intervention on symptom measures in this and other postnatal settings.

**Trial registration:**

Australian and New Zealand Clinical Trials Registry, ACTRN126220000354785. Registered 1 January 2022—retrospectively registered.

## Key messages about feasibility

What uncertainties existed regarding the feasibility?

The cognitive behavioural and art-based program (CB-ART) is a novel intervention for women in the postnatal period. This pilot was the first to evaluate CB-ART in this population. The study was designed to inform whether the CB-ART intervention was practical to implement at the Masada Private Hospital Early Parenting Centre (MPHEPC) as well as the capacity to integrate the CB-ART program into the existing MPHEPC schedule. We required an understanding of the acceptability of the CB-ART program to the women admitted and to ensure no harms were identified associated with participation in the program. The pilot was designed to provide preliminary data on the efficacy of CB-ART in this population and the value the CB-ART program could add to the MPHEPC program.

What are the key feasibility findings?

Evaluation of the CB-ART program indicated it was practical to integrate and implement into the existing MHPEPC program. There were modifiable contextual factors which limited demand for the CB-ART program, as it was scheduled at the same time as another popular program. The pilot informed the requirement for CB-ART to be scheduled at an alternate time in the existing program to limit this barrier to participation. The pilot also identified non-modifiable factors impacting participant engagement, such as the demands of the infant and fatigue experienced by the mothers. Evaluation of the participant’s response to the CB-ART program indicated it was an acceptable program with potential efficacy in this population group.

What are the implications of the feasibility findings for the design of the main study?

The pilot study indicates that the integration, implementation and structure of the CB-Art program is appropriate. It is a practical and acceptable intervention which was deemed beneficial by the participants. However, improvement in the scheduling of the CB-ART program is required to improve program demand. The limited efficacy testing conducted indicated the potential requirement for alternate, or more sensitive, quantitative measures to evaluate the efficacy of CB-ART.

## Background

The perinatal period covers the time from conception to 12 months’ postpartum. The transition into motherhood entails physical, emotional and psychological adjustments, necessitating re-evaluation of autonomy, personal liberty and identity. This can result in a degree of psychological disequilibrium and can confer an elevated risk of depression and anxiety [1]. The risk profile is exacerbated by a history of depression or anxiety, experience of physical or emotional birth trauma, postnatal pain, health-risk to the infant, maternal perinatal stress, insufficient social support, child-sexual abuse and inter-personal violence [2, 3]. A 2015 systematic review found the prevalence of antenatal and postpartum depression as estimated between 7 and 30% globally, with elevated risk in low-income countries [4]. Compromised maternal mental health has adverse ramifications for the women’s health and her infant’s health and development [2, 5, 6].

A review of the risk factors predisposing to the development of maternal mental distress identified the importance of early identification to implement support and intervention and the need for further research into the efficacy of nonpharmacological treatments [4]. A qualitative systematic review inclusive of 59 studies from diverse cultures concluded that ‘talking therapies’, built on empathic listening, was the treatment of choice of new mothers, with emphasis placed on the benefit of support groups [7]. The literature has shown that interventions that are focused on improved mother–child relationships [8, 9], culturally sensitive psychological therapies [10, 11] and improved social networks are required.

Cognitive behavioural therapy (CBT) is a short-term, goal-oriented psychotherapy that is well established as a first-line treatment for psychological distress, with particular efficacy in the management of anxiety and depression. The principles of CBT are based on the underlying premise that thoughts, feelings and behaviours are interrelated, whereby a change in one area leads to a change in the other domains. The aim of CBT is to modify emotional or behavioural problems by adjusting negative thoughts and attitudes and replacing them with more adaptive approaches [12]. With assistance from a therapist, edits in these distortions can result in a productive revision in perceptions leading to improved mood and positive behaviour change. There is good evidence for the utility of psychological therapies such as CBT in the perinatal period [13]. Evidence assembled in a Cochrane review [14] showed that psychosocial and psychological techniques are effective means to improve maternal mental health when pharmacotherapy during pregnancy and lactation may be contraindicated or not desired [4]. However, a limitation of CBT is the significant cognitive and verbal engagement required for abstract thinking in therapeutic cognitive exercises. This can be challenging for those with concrete thinking styles or lower verbal skills [15]. A systematic review on common help-seeking barriers in the perinatal period identified difficulty discussing emotions in traditional psychotherapeutic models and fear of the stigma associated with mental illness [7].

**Fig. 1:**
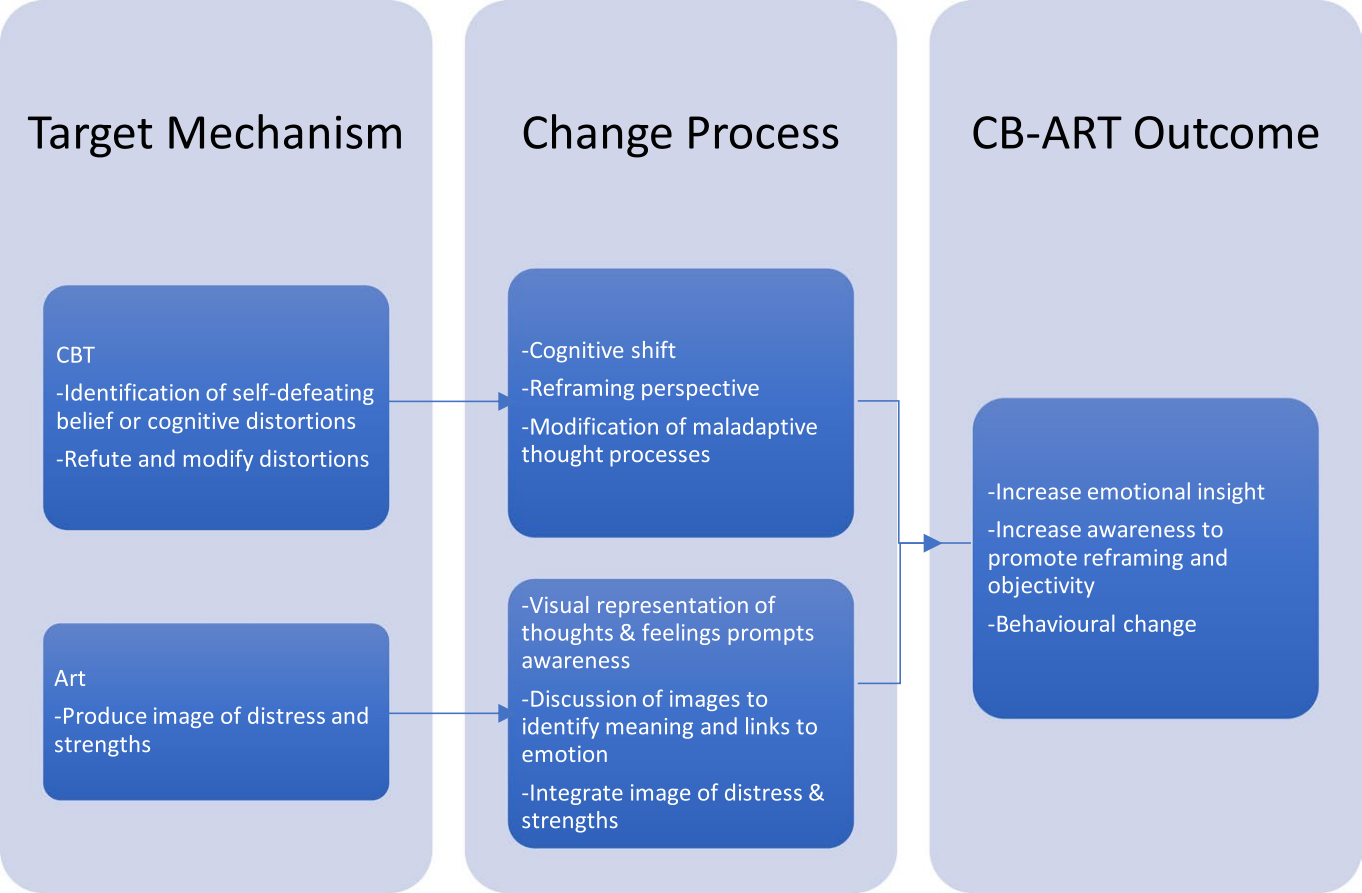
Proposed theory of change in CB-ART

A recent literature review on art therapy (AT) in the perinatal period indicated the utility of group-based AT to assist in the transition to motherhood with benefits in self-esteem, connectivity, self-reflection and improved mother–infant dyad relationships [16, 17]. AT is a creative form of psychotherapy which incorporates creative methods, such as drawing, sculpting or music, to assist the therapeutic process. AT is based on the premise that artistic expression occurs as a result of the convergence of cognitive and emotional processes that can facilitate healing [18]. Some people find emotions or feelings difficult to verbalise. AT provides the opportunity for participants to express feelings and somatic sensations by creating a visual representation of their psychological state. This enhances communication between client and therapist by creating a ‘window’ into their lived experience. The act of art creation also stimulates areas of the brain responsible for storage of visual memories [19] and assists recapture and reframing of memories in order to restructure them into a more productive framework [20]. It has been utilised successfully in a range of modalities including trauma and crisis therapy [21] and to mitigate pain associated with chronic illness [22]. The synthesis of AT techniques with CBT serves as an opportunity to address the potential barriers to talking-based therapy, whilst adding the therapeutic value of art-based interventions [17, 23] (Fig. 1).

Although previous studies have utilised art therapies or psychological therapies, few studies have reported on perinatal interventions that combined AT and CBT [17]. The cognitive behavioural and art-based intervention (CB-ART) is a novel treatment protocol that combines the theoretical approaches of AT and CBT in individual or group settings. It draws on cognitive behavioural interventions, centred around art-based activity, to incorporate symbolic, therapeutic creative based narratives with cognitive restructuring techniques to improve function, as described by the developers [23]. The results of the published protocol indicate the potential benefit of CB-ART during pregnancy and after birth. The aim of this pilot study was to assess the feasibility, acceptability and preliminary effectiveness of CB-ART, an art-based psychotherapy program, among women experiencing adjustment difficulties after giving birth who are admitted to a residential early parenting centre.

## Methods

### Study design and setting

The study was a single-centre, mixed methods pilot study to evaluate CB-ART for acceptability, feasibility, safety and preliminary efficacy. It was evaluated among women admitted to a 5-day residential early parenting service, Masada Private Hospital Early Parenting Service (MPHEPC), in Melbourne, Australia.

MPHEPC offers a 5-day residential early parenting admission for women and their infants up to 2 years of age. It caters to women experiencing infant feeding and sleep difficulties, irritability, reflux and colic, exhaustion, anxiety, problems in adjustment to parenting and postnatal depression. The centre is staffed by a multidisciplinary team that includes doctors, nurses and clinical psychologists. The treatment program addresses maternal and infant needs. Maternal physical health is assessed on admission and problems are treated or referred to a specialist practitioner. Psychoeducation normalises adjustment difficulties, provides strategies for successful adjustment to motherhood, and addresses infant behaviour and development. Participation in the program is associated with improvements in infant behaviour and maternal mood compared to pre-admission [24]. MPHEPC staff judged that CB-ART could be a useful addition to the MPHEPC program. The research was conducted in a collaboration between the CB-ART developers, the researchers and MPHEPC staff.

### Participants and recruitment

Inclusion criteria were woman admitted with her infant to the MPHEPC program. Recruitment and implementation of the CB-ART program took place over a 3-week period, during which time three separate CB-ART groups were conducted. Although CB-ART is for women in the postnatal period, one father requested an opportunity to join the study. Permission from the other group members was sought and granted.

### Intervention

The CB-ART intervention, implemented in the pilot, was adapted from the original CB-ART published protocol [23]. The original CB-ART protocol was developed by co-authors OS and JC, in collaboration with a team of specialist researchers in women’s health and intervention development. This team comprised of art therapists, psychotherapists and specialist researchers operating in women’s health and art therapy. The CB-ART approach has international evidence for use in a variety of adult populations including those experiencing chronic pain, depression and anxiety [22].

The original protocol detailed a six-session approach. This was adapted by the HB and JF, in collaboration with the original developers, into a two-session format in order to suit the MPHEPC 5-day residential program. The protocol guided the choice of selected materials as well as discussion prompts and processing of the art in session. It was offered over two, 1-h small group art and discussion sessions for 3–4 participants. Sessions were facilitated by a clinical psychologist (JF) and assisted by the first author (HB). New art materials were placed at the centre of a large oval table. Within easy reach of all participants were A3 sheets of white paper, oil pastels in at least ten colours, coloured pencils, grey lead pencils, charcoal and erasers. The sessions commenced with a brief introduction to confirm that the content of sessions was confidential, artistic capabilities were not needed, participants could retain or contribute their art and no assessment of the art was involved.

Participants were invited in the first session to draw an image, symptom or memory associated with a personal source of stress and, in the second session, associated with a personal source of strength or resource. Participants were given ten uninterrupted minutes to create an image on each occasion. These drawings were used by the psychologist to facilitate group discussion, beginning with the opening question ‘Tell me about this specific image, symptom or memory that you drew’. Responses were recorded to capture verbatim the specific clients’ wording or description of their piece and observation on the compositional elements of the image. Each participant was invited to talk about what had been drawn, and what they thought it meant to them, and then to respond to questions or comments from other group members. In the second session, participants were invited to respond to compositional elements of difference they observed between their own or others’ first and second drawings. Cognitive behavioural techniques were employed to assist participants to reframe thoughts and perceptions and to facilitate solution-focussed problem-solving.

### Measures

Quantitative and qualitative methods of data collection were used: a pre- and post-intervention questionnaire, including two standardised measures, the Short Profile or Emotional Competence (S-PEC) [25] and Edinburgh Postnatal Depression Scale (EPDS) [26]. Qualitative data were field notes documenting women’s discussion of their images in the CB-ART group and a post-intervention study-specific structured telephone evaluation interview. These datasets were each analysed thematically.

### Quantitative pre-and post-intervention questionnaire

#### Demographics

Self-report pre-implementation questionnaire included mother’s age, infant’s age, number of children, country of birth, relationship status, languages spoken, education, employment and health insurance status.

#### Short profile of emotional competence

Emotional insight, which refers to an individual’s capacity for identification, expression, understanding, regulation and use of their emotions as well of those expressed by others, was assessed using the validated S-PEC [25]. The S-PEC is a 20-item questionnaire with 10 items relating to intrapersonal emotional competence (EC) (e.g. ‘I do not always understand why I respond the way I do’) and 10 items related to interpersonal EC (e.g. ‘most of the time I understand why other people feel the way they do’). Responses are recorded on a 5-point Likert scale. It is adapted from the Profile of Emotional Competence [27]. The reliability was good (intrapersonal EC score *α* = 0.86; the interpersonal EC *α* = 0.89 and total EC *α* = 0.92) [25] when assessed among participants in the general adult population. The S-PEC has high internal consistency and composite reliability. Higher scores indicate greater emotional insight.

#### Edinburgh postnatal depression score

The EPDS is a standardised screening tool to detect women who have probable clinically significant symptoms of postnatal depression [26]. The EPDS is a 10-item questionnaire, with response options scored from 0 to 3 to generate a total score out of 30. A total score of ≤ 9 is indicative of the presence of none to minor symptoms of distress. These symptoms generally self-resolve. Scores from 10 to 12 indicate the presence of moderate symptoms of depression. Scores of 13 and above indicate a high likelihood of clinical depression (sensitivity 86%; specificity of 78%). EPDS has good reliability (0.88) and high internal consistency (*α* = 0.87) [26].

#### Post-intervention structured CB-ART evaluation

A study-specific questionnaire captured participants’ experiences, perceived personal impact and impressions of the CB-ART program each on a 5-point Likert scale.

### Qualitative

#### Field notes of discussion

Field notes were taken at each of the CB-ART sessions. Participants’ verbalisation of their images and the resultant discussion that emerged was documented verbatim. This was recorded in addition to the context and mood of the discussion.

#### Images created in CB-ART sessions

The drawings created by participants in the CB-ART sessions were collected or photographed at the end of each CB-ART session. Participants gave permission for the publication of their drawings. The images are linked to participants’ discussion points.

#### Post-intervention structured telephone interview

Follow-up interviews were administered, 1 week after discharge from MPHEPC. This involved a brief 10-min follow-up questionnaire with an opportunity for participants to give additional, unstructured evaluation feedback regarding their experiences of the program.

### Procedure

All women admitted to the MPHEPC over a 3-week period were approached by HB on day 1, shortly after admission, and invited to attend CB-ART sessions. It was emphasised that no experience of art-making was necessary for participation. Those who expressed interest in participation were given a letter of invitation and re-visited on day 2 of admission to confirm their interest. Women who agreed to participate were provided with a package of materials containing a plain language statement, an informed consent form, self-report pre-intervention questionnaire, contact details form and a withdrawal of participation form.

All forms were coded and contact details forms were stored separately from research data to ensure confidentiality. Participants’ completed questionnaires and signed consent forms were returned to the researcher immediately prior to the first CB-ART session. Informed consent included permission for the researcher to access medical records in order to document participants’ admission EPDS scores. Participants’ verbal permission was sought to allow field notes to be taken by HB during CB-ART sessions and for the researchers to retain drawings for anonymised inclusion in related publications.

Participants attended two CB-ART sessions, on days 2 and 5 of admission (7:30 p.m. and 2:30 p.m., respectively), and a 10-min structured telephone interview conducted by HB 1 week after discharge from the MPHEPC. The telephone interview included re-administration of the S-PEC and EPDS in addition to evaluation questions regarding the CB-ART program.

### Data coding and analysis

Data were analysed with SPSS software. Mean scores were calculated for total and subscale S-PEC and EPDS scores. Differences pre- and post-intervention scores were computed.

The CB-ART evaluation scores were collapsed from a 5-point Likert scale to a 3-point Likert scale: agree, neutral or disagree. Descriptive data were calculated and the data were tabulated to indicate the number of participants who endorsed each response option.

Field notes documenting participants’ discussions during the CB-ART sessions were transcribed. The text was analysed by HB using a thematic approach [28]. This is a method to identify, code, group according to theme and revise the data to guide interpretation. The initial stage established familiarity with the data in a naïve reading. Codes were then allocated. Themes were extracted and recorded in tabulated form, revised and then collated into coherent and discrete concepts. The data were linked to the corresponding image created in the CB-ART session and illustrative quotations selected and applied to each theme. The results of the three CB-ART groups are presented together.

## Results

Thirty-six women were invited to participate. Twenty-six expressed verbal interest; 8 later declined due to competing demands, such as conflicting activity scheduling (*n* = 7) and lack of interest in art (*n* = 1). Eighteen were provided with the participation package; 10 later declined participation due to unsettled infant (*n* = 5) or conflicting activity (*n* = 5). The remaining eight participated and provided full data. One father joined the group with his wife and provided complete data, except for the admission EPDS which were retrieved from MPHEPC records (total *n* = 8).

Characteristics of participants are detailed in Table 1. The participants were physically healthy, mean age of 32 years (range 28 to 39 years). They were predominantly first time parents (*n* = 6), all with infants under the age of 12 months. All participants had private health insurance, an indicator of social advantage, and were married. All but two participants were born in Australia (one was born in China and one in Brazil).

**Table 1:** Demographic details for participants (*n* = 9)

	Range	Number
Age	< 30	3
	30–35	4
	> 35	2
Infant age	≤ 4 months	1
	4 ≤ 8 months	4
	8–12 months	4
	≥ 12 months	0
First infant	Yes	6
	No	3
Relationship status	Single	0
	Partnered	0
	Married	9
Education level	TAFE/Certificate	1
	University	8
Country of birth	Australia	7
	China	1
	Brazil	1
Primary language	English	7
	Chinese	1
	Portuguese	1
Residential location	City/inner suburbs	3
	Outer suburbs	5
	Major regional city	1
Private health insurance	Yes	9
	No	0
EPDS	Baseline score below clinical range indicating few symptoms of probable depression (< 10)	2
	Baseline score 10–12 indicting moderate symptoms of depression	0
	Baseline score in clinical range indicating probable depression (≥ 13)	7

Three two-session CB-ART programs were conducted on consecutive weeks, each with an independent group of participants completing the program. Three participants were present in each group. All participants completed both sessions. No adverse events were reported.

### S-PEC and EPDS

Almost all participants (*n* = 7) met criteria for probable depression (EPDS ≥ 13), which is high compared with other samples of women admitted to MPHEPC [29]. S-PEC scores were similar to the normative sample [25] (Table 2).

**Table 2:** S-PEC and EPDS mean scores (*n* = 9)

Measure	Pre-intervention		Post-intervention		Comparison
	Mean (SD)	Range	Mean (SD)	Range	Mean (SD)
S-PEC total	3.42 (0.28)	3.05–3.9	3.47 (0.26)	3.15–3.8	3.42 (0.49) [25]
S-PEC interpersonal	3.36 (0.30)	2.8–3.8	3.41 (0.30)	2.9–3.8	3.52 (0.51) [25]
S-PEC intrapersonal	3.49 (0.37)	3.2–4.3	3.52 (0.32)	3.0–3.9	3.33 (0.62) [25]
EPDS	14.25 (4.49)^a^	5–21	8.11 (3.95)	2–15	11.9 (5.6) range 1–29 [29]
EPDS ≥ 13 (% on admission)	15.57 (2.70)^a^ [88% participants]	13–21	8.29 (4.54)	2–15	46.4% participants ≥ 13 [29]

^a^ (*n* = 8) father’s participant data missing

Comparison of mean pre- and post-intervention scores shows marginal improvement for S-PEC (emotional insight global, interpersonal and intrapersonal scores) and marked improvement for the EPDS scores (Table 2). Significance tests were not conducted because of the small sample size.

### CB-ART evaluation

The post-intervention evaluation data were grouped into feedback regarding the feasibility of CB-ART’s inclusion in the MPHEPC program, its acceptability and participants’ perceptions of its effectiveness. All nine participants provided evaluation information (Table 3).

**Table 3:** CB-ART program evaluation

Theme	Question	Agree	Neutral	Disagree	Quotes
Feasibility	The sessions were about the right length	8	1	0	“it would have been good if they were slightly longer” P1002
	The materials that were provided were appropriate	8	1	0	“all positive” P1018
	The CB-ART sessions were easy to fit into my schedule at Masada	8	0	1	“it’s hard having it scheduled against the meditation group” P1019
	I think two sessions were sufficient to achieve the goals	8	1	0	“I would have liked more sessions, I really enjoyed it” P1009 “I think three smaller sessions with more focus on drawing would have been more effective” P1015
Acceptability	The drawing sessions were enjoyable	9	0	0	“it was a good way to relax” P1009
	The group was well facilitated	8	1	0	“It was a very safe and comfortable environment” P1013
	I found it easy to do the drawings	5	3	1	“drawing is a good conversation starter, but the drawing didn’t always continue. It would be nicer if there was more opportunity to draw throughout the session with more drawing directives” P1015
	I attended the group because I thought it would be helpful to me	6	2	1	“I have perceived the session to be more art focused” P1002
	I have personal experience of drawing or other art-making	1	1	7	“do not have a personal experience of art making in this kind of context” P1002
	There were some aspects of the program that were unpleasant	1	0	8	“it was unpleasant to discuss feelings that make you sad, but the program was not unpleasant in itself” P1002
	I would consider participating in a similar program in the future	9	0	0	“I would have liked more sessions, I really enjoyed it” P1009
	I would recommend the CB-ART program to others in similar circumstances	9	0	0	“I went because another mother wanted me to come with her, but I’m really glad I went in the end. I wish more people had took up the opportunity” P1019
Effectiveness	Participating in the group with other women was helpful	8	1	0	“I have felt better in this environment among people going through the same experience” P1019
	Drawing is a useful means of helping people to name their thoughts	8	1	0	“putting the focus on drawing rather than eye contact made it easier” P1013
	I think drawing helped participants to recognise their strengths	5	4	0	“the drawing took the pressure off when talking about very personal topics” P1013
	I found drawing the most useful part of the program	3	3	3	“the drawing part was the most fun, that was the reason I signed up to the program” P1002
	The group discussion was helpful	8	1	0	“The use of drawing to help people name thoughts and feelings was a new concept, but the discussion was very helpful” P1002
	I found the group discussion the most useful part of the program	8	1	0	“I didn’t think the program needed the drawing component” P1018
	I think the program helped me to identify my thoughts	9	0	0	“it felt really good and positive. Brought up many good things to think about” P1018
	I have continued to use drawing as a means of expressing my thoughts & strengths	1	4	4	“I don’t have much time to draw in the home environment” P1013

#### Feasibility

Almost all participants found the sessions easy to fit into their schedule at MPHEPC (*n* = 8) and thought that two CB-ART sessions were sufficient to achieve the goals (*n* = 8).

#### Acceptability

Overall, participants evaluated the sessions as enjoyable (*n* = 9) and well facilitated (*n* = 8). No negative feedback was reported. Few reported having any prior artistic experience (*n* = 1). All participants would consider participating in a similar program in the future (*n* = 9) and would recommend the CB-ART program to others (*n* = 9).

#### Safety and efficacy

Most of the participants (*n* = 8) identified the presence of other participants as positive. Almost all (*n* = 8) identified drawing as a useful means to help identify thoughts. Most participants found the benefit of the group discussion to be helpful (*n* = 8). All participants expressed the view that CB-ART helped them to identify their thoughts (*n* = 9).

### The CB-ART sessions

Analysis of the discussion and art generated by participants in the CB-ART sessions revealed a series of themes (Table 4).

**Table 4:** Images and quotes by participants in the CB-ART program

Participant	Group	Session 1 (S1)	Session 2 (S2)	Quotes
P1001	1			*“I feel like my heart is torn between the two countries. I fear what I am missing out on when I am not there” (S1)* *“Before I had blamed others that I was unhappy, but now I see that everybody is there and wants to help, they just don’t know how” (S2)*
P1002	1			*“I feel like everything’s pent up in my head and I can’t get control” (S1)* *“I associate horses with feeling strong, when I was riding horses I felt strong” (S2)*
P1005	1			*“I guess it’s symbolic. I often keep things very close and am quite a guarded person. I don’t give a lot of myself early on in a setting” (S1)* *“Without structure, you start to feel like you lose control” (S2)*
P1008	2			*“You move apart and you don’t have time to do those regular easy things” (S1)* *“It’s like a time from a completely different life. I’m yearning for the hustle and bustle of the city rather than the loneliness of an empty house” (S2)*
P1009	2			*“It’s a bit like spring, a time for new beginnings”(S1)* *“With mothering I feel an inner strength. This was me before motherhood, a bit wishful thinking and a bit lost” (S2)*
P1013	2			*“It’s like being sucked through a vortex” (S1)* *“I’m still looking for something exciting, how I can make my big contribution to the world, but it’s on hold at the moment” (S2)*
P1015	3			*“I work well with structure and I find it really difficult when my partner doesn’t stick to the structure” (S1)* *“I think I am the creative parent and I feel good about that. I think I feel particularly strong about the playing part” (S2)*
P1018	3			*“My husband feels like he’s failing because he can never meet my standards” (S1)* *“My baby is a smiley, giggly baby always looking around and trying new toys” (S2)*
P1019	3			*“This is the crown I’m wearing and this is my brain that’s all scrambled underneath, the crown is the front that I put on the part of myself that I show the world that looks composed” (S1)* *“At work I feel more confident, like a different person, there’s a level of control in the office I can’t achieve at home” (S2)*

#### Identity formation

Participants expressed some turbulence in their adjustment to the new identity required by the maternal role. There was expression of loss of prior interests, in particular the reduction in capacity for spontaneity and sense of independence. With this came a sense of re-alignment of their life around the baby, entailing re-ordering the hierarchy of needs, in which individual needs were placed secondary to their caregiving requirements. Some participants expressed a limited sense of validation for their adjustments because newborn babies are not yet able to provide positive feedback.

‘I feel like I don’t get much positive affirmation from my baby. She’s too young to smile yet and I really need that positive feedback that what I am doing is right’ P1013

‘What we miss is the ability to be spontaneous in general. To make it work, it’s not spontaneous anymore’ P1015

Despite the difficulties expressed in this adjustment to their identity as a parent and individual, there was acknowledgment of the capacity for positive transformation through the new role. This encompassed a sense of growing confidence as a mother and pride in the healthy development of their infant.

‘It’s a bit like spring, a time for new beginnings. I feel like with motherhood I’m finally getting that confidence back… With mothering, I feel an inner strength.’ P1009

#### Agency

The dynamics of transition from individual to parent included challenges to participants’ sense of agency and control. The perceived lack of control over the environment and internally was often difficult for participants to articulate. One participant’s image (P1019) indicated that the composed façade she presented to the world hid the internal chaos she was experiencing.

‘This is the crown I’m wearing and this is my brain that’s all scrambled underneath. The crown is the front that I put on, the part of myself that I show the world that looks composed.’ P1019

Participants spoke of the sense that their world had shrunk and that the fatigue impaired their resilience. There were many expressions of difficulties achieving and maintaining time for self. This was often contrasted with prior experiences, such as in a workplace capacity. Participants expressed difficulties applying the same techniques that had been essential to their professional success to their early parenting role.

‘At work I feel more confident, like a different person… There’s a level of control in the office that I can’t achieve at home.’ P1019

‘My world is so much smaller now, so I don’t have a sense of perspective to deal with things with the resilience that I used to’ P1008

Participants reflected on the skills they were gaining to exert some agency over their environment. The capacity to create a sense of structure was associated with a sense of agency and empowerment.

‘I need the predictability and the routine to be able to find time for myself’ P1019

#### Connection

The theme of social connection included discussion of the sense of relief associated with capacity to share the burden with others. There was a sense of joy and connectedness, when support was forthcoming from partners, and in the shared experiences of others, which normalised the motherhood experience. However, there was also the potential for friction, in particular with the intimate partner and family. Participants spoke of tension associated with conflicting parenting styles. This included commentary on perceived criticism, competition and methods of negotiating differences.

‘before I had blamed others that I was unhappy’, ‘I now see that [family] is there and wants to help, they just don’t know how’ P1001

‘I intimidate him because I do everything to perfection… He feels like he’s failing because he can never meet my standards.’ P1018

Some participants identified feeling abandoned by their partners, with long periods home alone with their infant. There was an acknowledgement of the difficulty managing differing cultural expectations, entailing differing perceptions of approaches to parenting and maternal and paternal roles. Geographical displacement from family was regarded as causing difficulties establishing a supportive network.

‘Before when my husband wanted to go out with his friends I would make him feel bad about it, why don’t you want to stay home with me and our son? But I see that this is hurting our relationship’ P1001

#### Environmental factors

Many external social factors were identified that influenced participants’ experiences and perceptions of early parenthood. Many spoke of the shift from their prior expectations to the experienced reality, with a sense of loss and betrayal caused by the unrealistically positive and ‘intuition driven’ expectation of motherhood presented by society.

‘I had always had a somewhat romantic view of motherhood and that I’d be guided by intuition, no one tells you that might not be the case.’ P1013

The sense of increasing societal pressure to juggle multiple roles, in addition to motherhood, was commonly expressed. This was discussed also in reference to the difference in expectation between cultures. A number of participants expressed feeling stuck between cultural worlds and expectations.

‘It’s [the hot air balloon] hanging over the water stuck between the two countries’, ‘It could pop at any moment’. P1001

‘There’s a lot of pressure to be a working mother, as if being a mother isn’t enough or that you aren’t enough if you don’t need to search for more’ P1009

There was acknowledgement of the constraints on free expression of adjustment difficulties. Many expressed the guilt experienced when they compared themselves with others experiencing fertility problems.

‘I feel like I have no entitlement to complain, when you look at all the other mothers that can’t conceive’ P1009

## Discussion

This pilot study is the first to evaluate the feasibility, acceptability and preliminary efficacy of CB-ART, a promising therapeutic intervention for women experiencing problems with infant care and adjustment difficulties, in a new, highly relevant setting. CB-ART was found to be a feasible, acceptable and potentially effective addition to an established Australian early parenting centre. The study contributes to the emerging literature investigating the use of art and cognitive behavioural practices to improve mental health among women in the perinatal period. CB-ART used in this setting confirmed the value of combining art-based approaches with CBT in this population [23]. Participation in the CB-ART program was associated with a clinically meaningful reduction in the symptoms of postnatal depression. Although establishing causality is not possible in a before and after study design, this finding does suggest that the intervention is safe. There was a marginal, improvement in emotional insight, as assessed by the S-PEC in the short term. This, in conjunction with the qualitative evidence that the program was highly evaluated by the participants. They identified that the artistic activities prompted rich discussion, moderated by an experienced clinical psychologist, indicating probable beneficial therapeutic effects. We also believe that the ability to draw an inner stress-related state on the paper as an image enhances the capacity for cognitive restructuring, for seeing things in perspective and to re-frame their experiences in constructive ways.

### Feasibility

The number of participants was lower than expected, although this permitted small group sizes, regarded by participants as valuable. Several factors influenced recruitment. The demands on women’s time during the MPHEPC program are high. The program scheduling coincided with a popular meditation group, which obstructed women’s capacity to participate. These contextual factors are modifiable and therefore do not preclude appropriate scheduling of CB-ART in the MPHEPC program in future. CB-ART offered as part of usual MPHEPC practice without the somewhat burdensome research component may also facilitate participation. Non-modifiable factors which affected participation in the CB-ART group included competing demands of the baby, presence of other family members and work requirements. Most women admitted to the unit are experiencing profound fatigue and some may have had insufficient available psychological resources for the perceived emotional and social contribution required in the CB-ART sessions. However, these factors apply potentially to all opportunities for engagement in this population.

### Acceptability

The CB-ART theory of change demands engagement with emotionally arousing material. The positive evaluation of the pilot program suggests that the presence of a skilled facilitator was vital to eliciting content and mediating group discussion whilst containing participants’ emotions and reactions to the other group members. Participants’ reflections also suggest significant value in the presence of a facilitator to assist in reframing thoughts and guide solution-focussed problem solving to promote well-being. The group format was also regarded as central to the shared reflective experience and sense of connection, which is consistent with similar successful programs [17].

### Safety and preliminary effectiveness

Evaluation of the participants’ pre-intervention and post-intervention EPDS scores indicates a marked improvement in the symptoms of postnatal depression. However, the CB-ART was being offered as part of the MPHEPC program, participation in which is also associated with reductions in EPDS scores of similar magnitude [24]. In this uncontrolled pilot study, improvements in symptoms are unlikely to be attributable to the CB-ART program alone. However, the near universal improvement in EPDS score, in the context of the unanimous positive evaluation feedback, suggest that the CB-ART program is safe and that the small group discussions were sufficiently well moderated by the facilitator to maintain and promote participants’ psychological well-being.

### Emotional insight

Emotional insight describes an individual’s capacity to identify, understand, express and regulate their emotions, as well as interpret and adjust to those around them. It is an important quality for successful adaptation to the surrounding environment. The factors which influence insight are complex and include developmental environment, personality factors and reflective capacity [30]. This occurs generally over time and experience. The mean S-PEC baseline score for emotional insight was consistent with the population norm. The post-intervention score improvement in the MPHEPC setting is unlikely to be clinically significant. It suggests that greater exposure to the proposed mechanisms of change in emotional insight over a longer period, as described in the original CB-ART protocol, which was recommended to be implemented between 4–6 sessions, may be warranted [23]. It is also possible that, although a validated measure, the S-PEC does not adequately measure the targeted change mechanism or is not sensitive to change over a short time interval. This suggests the need to pilot test an alternate instrument prior to a large-scale trial.

The discussion themes that arose in the groups’ guided reflection on the images created during the CB-ART sessions provided valuable insights about the prominent concerns of women experiencing adjustment difficulties at this life stage. They related to social connection, identity and agency as well as the influence of social and environmental factors. This, coupled with the positive perception of the CB-ART program to facilitate the process of reframing thoughts and engaging in problem solving, is encouraging. CB-ART was experienced as safe and non-threatening and enabled women to make new discoveries about their emotions and psychological needs.

Participants were already admitted to the MPHEPC program. It involves knowledge and practice in soothing and settling techniques, developing understanding of the baby’s needs for sleep, play and nutrition, which are provided by highly supportive and skilled staff [1]. This supportive milieu, combined with the shared peer to peer experiences of being admitted to a service with up to 20 mothers and infants, may have supported participants’ capacity to engage meaningfully with the CB-ART group intervention.

## Strengths and limitations

A strength of this study lies in the success with which this complex intervention was integrated into an existing short admission program in a hospital setting and systematic recruitment of the sample was enabled. Despite the fact that CB-ART was novel in MPHEPC, staff were supportive and women were willing to participate within the limitations on their time and other commitments. The study is further strengthened by the availability of an experienced clinical psychologist group facilitator, who provided emotional containment, consistent delivery of the CB-ART intervention and management of the resulting discussion of sensitive material. Other strengths include the use of standardised measures to evaluate psychological outcomes, structured interview guide and independent cross checking of data.

However, the small sample size precluded the application of statistical tests and limits the confidence with which conclusions can be made. The lack of a control condition makes it difficult to conclude that any observed improvements in functioning resulted from participation in CB-ART alone. Carefully recorded field notes were used rather than audio-recording to avoid detracting from the spontaneity of the conversation. However, analysis of verbatim transcripts would have enabled greater depth of thematic or discourse analysis and perhaps have provided greater insight into the mechanisms of action of CB-ART. Finally, an alternative measure of emotional insight needs to be considered. The S-PEC as an indicator of emotional insight in this context may require reconsideration. It may not have been sensitive enough to detect significant change over the short time course of the pilot. A final limitation of the study was that the investigator, HB, assisted in the delivery of the CB-ART program as well as conducted the evaluation interviews, which may have constrained participants’ willingness to express negative evaluations of their experiences.

## Conclusion

The CB-ART program was well accepted by women and deemed a feasible addition to the MPHEPC program. There were no negative impacts expressed. This art-based therapy shows promise in this setting and perhaps for other women encountered in other medical or therapeutic settings, navigating a difficult transition to new motherhood. The discussion themes provide a powerful insight into the level of peer-to-peer emotional sharing and reflection achieved in the CB-ART sessions, suggesting that the CB-ART method has promise in combination with the MPHEPC program and potential value in this wider population. This pilot study is the first step in the evidence-building trajectory. It has provided important information to inform to the protocol for a properly powered randomised effectiveness trial to provide better understanding of the effectiveness of CB-ART and its potential value for women in assisting in their adjustment to motherhood.

## Data Availability

The datasets used during the current study are available from the corresponding author on reasonable request. The trial was retrospectively submitted to the Australian and New Zealand Clinical Trials Registry.

## Abbreviations

AT: Art therapy
CB-ART: Cognitive behavioural and art-based intervention
CBT: Cognitive behavioural therapy
EC: Emotional competence
EPDS: Edinburgh Postnatal Depression Scale
MPHEPC: Masada Private Hospital Early Parenting Centre
S-PEC: Short Profile of Emotional Competence
